# A novel lactylation-related gene signature to predict prognosis and treatment response in lung adenocarcinoma

**DOI:** 10.3389/fonc.2025.1549724

**Published:** 2025-03-14

**Authors:** Hongyi Zhang, Yihao Liu, Xuanguang Li, Chen Ding, Chunqiu Xia, Hua Huang, Hongyu Liu, Jun Chen

**Affiliations:** ^1^ Department of Lung Cancer Surgery, Tianjin Medical University General Hospital, Tianjin, China; ^2^ Department of Thoracic Surgery, Renji Hospital, Shanghai Jiaotong University School of Medicine, Shanghai, China; ^3^ Tianjin Key Laboratory of Lung Cancer Metastasis and Tumor Microenvironment, Tianjin Lung Cancer Institute, Tianjin Medical University General Hospital, Tianjin, China

**Keywords:** lactylation-related genes, lung adenocarcinoma, immune cell infiltration, single-cell RNA sequencing, bulk RNA sequencing

## Abstract

**Background:**

Lactylation, a novel post-translational modification, has emerged as a critical regulatory mechanism in various biological processes, including tumor progression. However, its role and associated gene signatures in lung adenocarcinoma (LUAD) remain unclear.

**Methods:**

RNA sequencing data of LUAD patients were obtained from The Cancer Genome Atlas (TCGA) and Gene Expression Omnibus (GEO) databases. Unsupervised clustering was used to identify lactylation-related genes. A risk prognostic model was constructed using least absolute shrinkage and selection operator regression analysis and subsequently validated. A nomogram was then employed to optimize the clinical applicability of the risk score. Additionally, various algorithms were used to explore the relationship between the risk score and immune infiltration levels, with model genes analyzed based on single-cell sequencing. The effects of RCCD1 knockdown on LUAD cell proliferation and migration were evaluated through CCK8 and transwell assays.

**Results:**

Higher risk scores were associated with poorer overall survival prognosis. Immune analysis revealed that the risk score may play a role in regulating the tumor microenvironment. Additionally, these risk scores were found to be associated with chemotherapy drug sensitivity. A series of experiments further demonstrated that RCCD1 promotes LUAD cell proliferation and migration *in vitro*.

**Conclusion:**

This study highlights the critical role of lactylation-related gene signatures in LUAD and their association with immune cell infiltration, providing insights into potential therapeutic targets and biomarkers for clinical application.

## Introduction

Lung adenocarcinoma (LUAD) is the most common histological subtype of non-small cell lung cancer (NSCLC) (1, 2). Despite advancements in surgical techniques, targeted therapies, and immunotherapies, LUAD remains a leading cause of cancer-related mortality (3). Therefore, there is an urgent demand for the discovery of new biomarkers to enhance early detection and treatment outcomes in LUAD.

Post-translational modifications (PTM) are crucial regulators of protein function, stability, and interactions, playing essential roles in cellular processes and disease development (4–6). One such modification, lactylation, is a recently discovered PTM involving the addition of lactyl groups to lysine residues on histones and non-histone proteins (7–9). This modification is primarily associated with intracellular metabolic changes. Unlike acetylation and methylation, which are commonly linked to the regulation of gene expression and chromatin remodeling, lactylation is influenced by the cell’s metabolic state, particularly under conditions such as hypoxia or altered metabolic pathways. This modification regulates protein function by altering protein-protein interactions, stability, and cellular localization, thereby playing a crucial role in processes such as metabolic reprogramming, immune responses, and tumor progression. Although research on lactylation is still limited, as an emerging post-translational modification, its potential role in disease mechanisms, particularly in cancer and metabolic disorders, is receiving increasing attention (10–12). Recent studies have revealed the significant roles lactylation may play in these processes. For instance, lactylated Apolipoprotein C-II has been shown to induce resistance to immunotherapy by promoting extracellular lipolysis in NSCLC (13). Additionally, LKB1 has been found to induce cellular senescence and apoptosis *in vitro* and *in vivo*, with LKB1 inhibiting telomerase activity and promoting cellular senescence via histone lactylation in lung adenocarcinoma cells (14). Lactate-induced lactylation of IGF1R protein has also been shown to promote lung cancer cell proliferation and metabolic reprogramming (15). Furthermore, lactate enhances the stability of the IGF1R oncogene protein, thereby promoting glycolysis and lactate production, creating a positive feedback loop. Despite these promising findings, the functional roles and clinical significance of lactylation-related genes in LUAD remain poorly understood, highlighting the need for further investigation into their potential as therapeutic targets and prognostic markers in LUAD.

The tumor microenvironment (TME) plays a pivotal role in the progression of LUAD, influencing tumor growth, metastasis, and response to therapy (16, 17). Immune cell infiltration, particularly the recruitment and activity of subsets such as T cells, macrophages, and dendritic cells, is a critical determinant of antitumor immunity and the efficacy of immunotherapies (18). Recent studies have highlighted the impact of metabolic alterations in the TME, including lactate accumulation, on immune cell function and polarization (19, 20). For example, SLC2A1 has been identified as a prognostic biomarker in lung squamous cell carcinoma (21). Cancer-associated fibroblasts may reduce the effectiveness of PD-1/PD-L1 blockade in immunotherapy through glycolysis-induced lactate accumulation via LOX (22). However, the relationship between lactylation-related genes and the immune microenvironment in LUAD remains unclear. This warrants further investigation to explore how lactylation modulates immune cell dynamics and influences the TME, potentially offering new insights into LUAD treatment strategies.

In this study, we investigated the role of lactylation-related genes in LUAD and established a lactylation-related gene (LRG) model. The identification of lactylation-related gene signatures with prognostic and immunological significance may provide new avenues for personalized treatment in LUAD, ultimately improving patient outcomes and deepening our understanding of this complex disease.

## Materials and methods

### Data collection and processing

The relevant transcriptomic data, mutation data, and clinical data were sourced from platforms such as The Cancer Genome Atlas (TCGA) and the Gene Expression Omnibus (GEO), including datasets GSE31210 and GSE72094. Data preprocessing included quality control and normalization, after which the three datasets were merged. The clinical information of the patients is in **Supplementary Table S1**.

### Identification of lactylation-related genes

Lactylation-related genes (LRGs) refer to those genes whose expression is either directly regulated by lactylation or involved in the cellular processes modulated by lactylation, based on findings from previous studies in the field. All LRGs in this study were collected from previously published studies. After removing duplicate genes, a total of 332 genes were identified, which are listed in **Supplementary Table S2**.

### Consensus lustering analysis

We used consensus clustering analysis to identify different subgroups of LUAD patients based on the expression patterns of LRGs. The k-means clustering algorithm was applied to the expression matrix of lactylation-related genes.

### Development of prognostic model

We performed a multivariate Cox regression analysis and split the LUAD patient cohort into two equal groups: a training set (n = 553) and a test set (n = 553). Key genes and their corresponding regression coefficients were selected using LASSO Cox regression. A risk score was calculated by applying the standardized expression values of the identified genes and their respective coefficients, following the formula: Risk Score = ∑ (Gene Expression × Coefficient). Based on the median risk score, patients were categorized into low-risk and high-risk groups. The performance of the model was assessed using receiver operating characteristic (ROC) curves.

### Drug sensitivity analysis

The “pRRophetic” R package was employed to calculate the half-maximal inhibitory concentration (IC_50_) values for drugs in LUAD (23).

### Exploration of the tumor immune microenvironment

The ESTIMATE scores were calculated using the “estimate” R package. Single-sample gene set enrichment analysis (ssGSEA) was conducted to quantify the proportion of immune cells within each sample.

### Establishment of prognostic scoring system

Based on the model scores, we developed a LRGs prognostic model, and combined it with clinical parameters (such as age, stage, and gender) to create a nomogram scoring system. The predictive accuracy of the scoring system was evaluated using calibration plots.

### Single-cell RNA sequencing analysis

Single-cell RNA sequencing data from 11 LUAD samples were obtained from the GSE131907 dataset. To analyze the cell populations, we performed clustering using the Seurat R package. Prior to clustering, batch effects were corrected to ensure reliable results. Cells were filtered based on specific criteria: those with fewer than 500 or more than 6000 unique feature counts, as well as cells with more than 20% mitochondrial gene expression, were excluded. After normalizing the data, we conducted non-linear dimensionality reduction using t-SNE with the default settings. Subsequently, cluster-specific biomarkers were identified.

### Cell culture and transfection

The NSCLC cell lines A549 and PC9 were obtained from the American Type Culture Collection (ATCC, Manassas, USA) and cultured in DMEM (Biological Industries, USA) supplemented with 10% FBS (Gibco, USA). Lipid-based transfection reagents were used to transfect target genes or siRNA according to the manufacturer’s instructions. Cells were collected for subsequent functional assays. Transfection was performed with siRNA targeting RCCD1 or control siRNA (Ribobio, China) and Lipofectamine 2000 (Invitrogen, USA).

### Quantitative reverse transcription polymerase chain reaction

Quantitative real-time PCR (qRT-PCR) was employed to assess the expression of RCCD1 in LUAD cell lines. qRT-PCR was carried out using SYBR Green PCR Master Mix, with GAPDH serving as the internal control for normalization of gene expression. Fold changes in gene expression were determined using the 2^−ΔΔCT method. The mRNA primer sequences used are shown as below: 5′-AGGGCCTAGTCATGGCTGAG -3′ and 5′-GGCTTCCCTTGCGACAGTC-3′ for RCCD1.

### CCK8 cell viability assay, colony formation assay, and transwell migration assay

The CCK8 assay was used to evaluate cell viability. Cells were seeded in 96-well plates and treated with si-RCCD1 or si-control, with absorbance measured at 450 nm after 24, 48, and 72 hours. Colony formation assay, cells were seeded in 6-well plates and cultured for 14 days, followed by crystal violet staining and counting. The transwell experiment was performed without a matrigel coating, and cells were stained with crystal violet after 48 hours.

## Results

### Analysis of molecular subtypes related to LRGs

**Figure 1:**
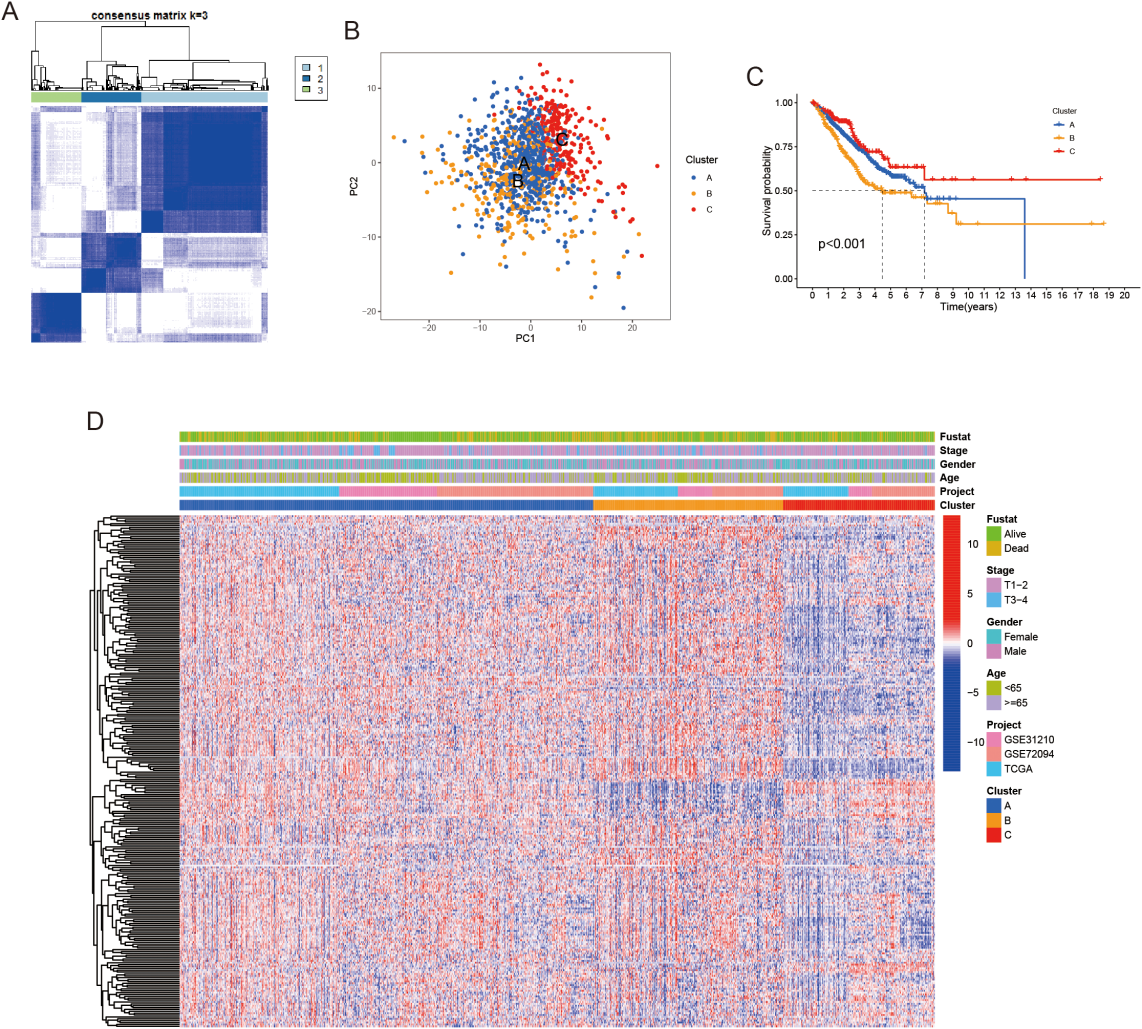
Unsupervised clustering was performed to identify lactylation-related genes. **(A)** LUAD patients were classified into three molecular clusters using a k = 3 approach. **(B)** Principal Component Analysis (PCA) revealed notable expression differences of lactylation-related genes across the three subtypes. **(C)** Kaplan-Meier survival curve demonstrated significant overall survival differences between the three clusters. **(D)** A heatmap displayed differences in lactylation-related genes expression and corresponding clinicopathological features across the three molecular clusters.

To investigate the molecular subtypes associated with LRGs, we conducted unsupervised clustering analysis using RNA-seq data from the TCGA and GEO databases. This approach enabled the identification of three distinct molecular subtypes in LUAD, with the optimal number of clusters determined as k = 3 based on the consensus cumulative distribution function curve (**Figure 1A**). These subtypes were labeled as clusters A, B, and C, reflecting differences in their underlying gene expression profiles. Principal component analysis was subsequently performed, highlighting the distinct molecular signatures driven by the expression of LRGs (**Figure 1B**). Notably, patients in cluster B exhibited significantly shorter overall survival (OS), (**Figure 1C**). In addition, we examined the expression levels of these LRGs in relation to the distribution of each molecular subtype and clinicopathological features, which are visually represented in **Figure 1D**. We found that some genes were significantly reduced in cluster C, which underscores the heterogeneous nature of LUAD and suggests that these molecular subtypes may reflect distinct tumor biological behaviors and patient outcomes. These findings provide important insights into how LRG expression may influence tumor progression and prognosis in LUAD.

### Tumor immune microenvironment analysis in different molecular subtype groups

**Figure 2:**
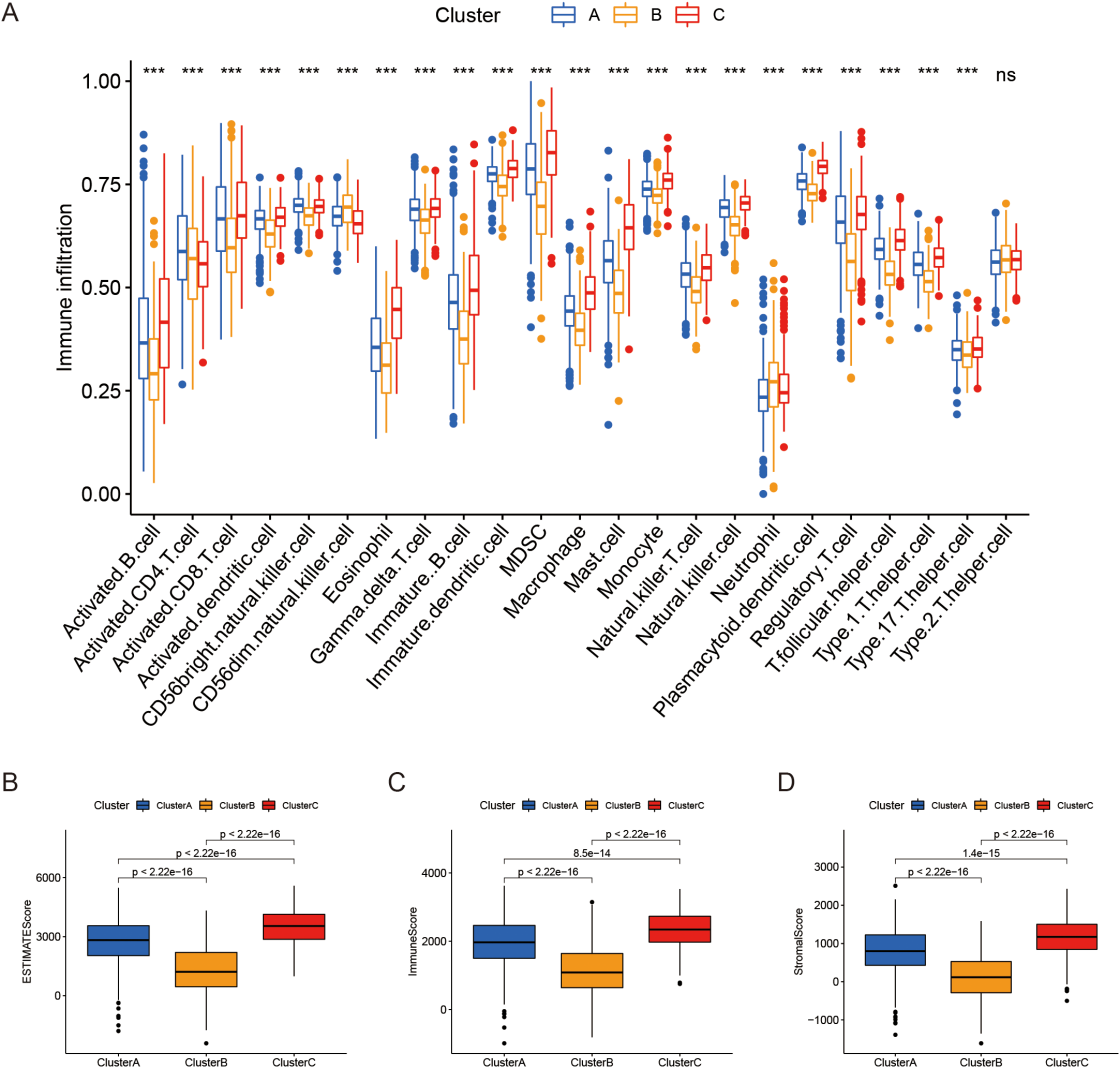
Comparison of tumor microenvironment properties across subgroups. **(A)** The proportion of immune infiltrating cell components across the three clusters. **(B-D)** Boxplots illustrating the differences in ESTIMATE score, immune score, and stromal score among the three clusters. ***P < 0.001, ^ns^P ≥ 0.05 compared to the control group.

The analysis of the tumor immune microenvironment across the molecular subtypes revealed significant differences in immune cell composition, which further highlights the complexity of LUAD. Notably, cluster C exhibited significantly highest levels of activated B cells, activated CD8+ T cells, and macrophages et al, which are often associated with an active immune response (**Figure 2A**). Additionally, patients in cluster C demonstrated markedly highest ESTIMATE scores, immune scores, and stromal scores, all of which are indicative of a more immunologically active tumor microenvironment and highest levels of stromal and immune cell infiltration (**Figures 2B–D**). These factors were positively correlated with a longest survival time, further supporting the notion that a more robust immune presence may enhance tumor control and improve prognosis. In contrast, the reduced immune infiltration and lowest ESTIMATE scores in cluster B were associated with the poorest survival outcomes, suggesting that immune evasion and lack of immune response may be key drivers of the aggressive nature of this subtype. These findings indicate that LRGs may play a pivotal role in shaping the immune landscape of LUAD.

### Establishment and verification of risk model based on differentially expressed genes

**Figure 3:**
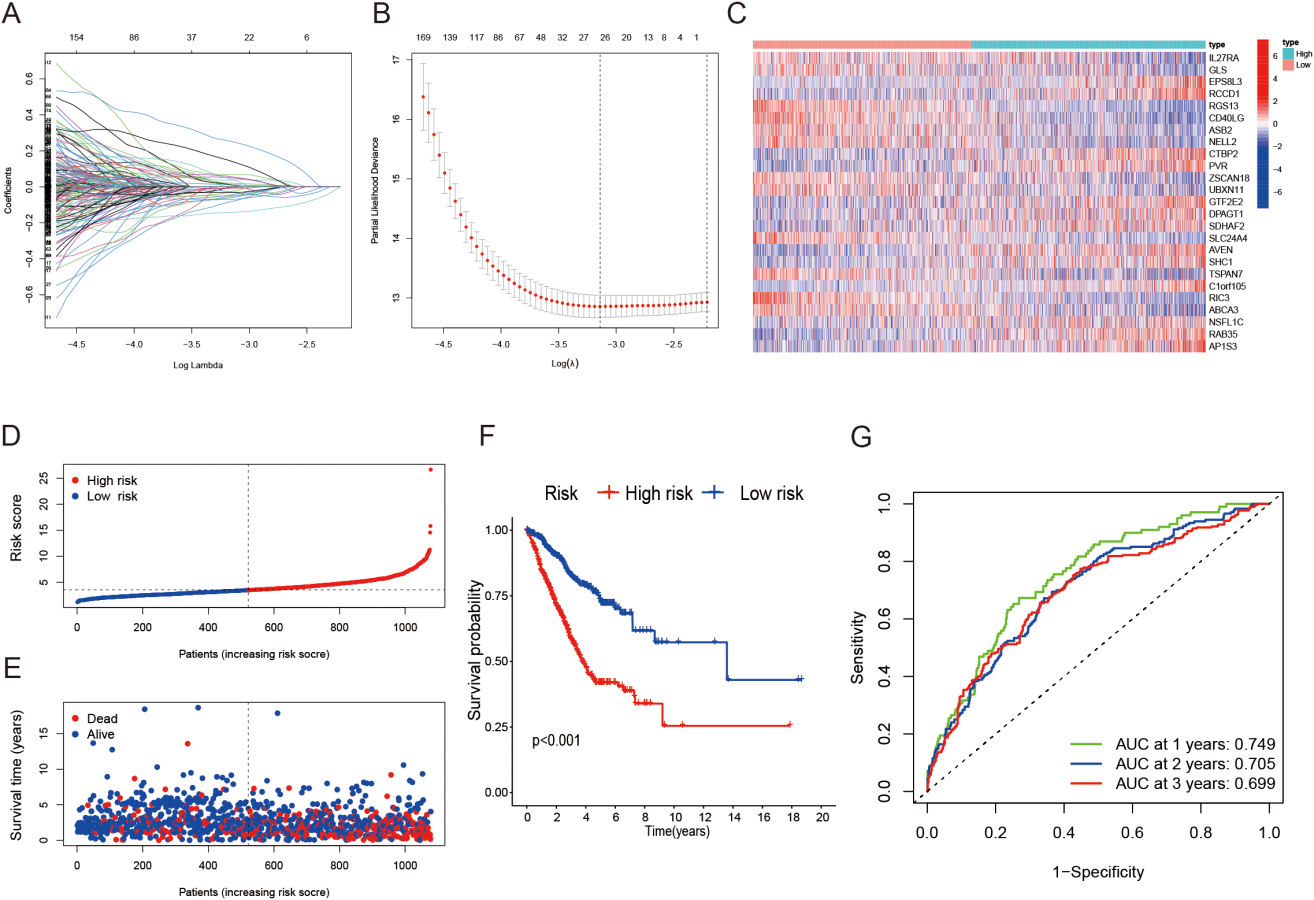
Construction of a lactylation-related prognostic model. **(A, B)** Determining the optimal number of factors using LASSO regression analysis. **(C)** Heatmap illustrating the 25 genes included in the model alongside clinical features. **(D, E)** Distribution of the risk score based on survival status and survival time in training cohort. **(F)** Kaplan-Meier survival curves showing overall survival for patients categorized into different risk groups. **(G)** ROC curves assessing the sensitivity and specificity for 1-, 2-, and 3-year survival predictions in the training cohort.

To investigate the potential biological behaviors of the LRG subtypes, we first identified subtype-specific differentially expressed genes (DEGs) using the limma package in R. Based on the DEGs identified across clusters A, B, and C, a risk prognostic model was developed. The patient cohort was randomly split into two groups at a 1:1 ratio, creating a training set and a validation set. The LASSO regression method was applied to build the model (**Figures 3A, B**). we developed a risk model in the training set, which demonstrated significant prognostic value. This model consists of 25 genes, with **Figure 3C** illustrating the expression levels of these 25 genes in the high- and low-risk groups. Higher risk scores were associated with poorer OS in LUAD patients (**Figures 3D–F**). Moreover, ROC curve analysis further confirmed the strong prognostic value of the model, highlighting its potential utility for clinical prognostic prediction (**Figure 3G**). To further validate the robustness of our model, we performed validation using the validation set, which yielded consistent and promising results (**Supplementary Figure S1A-E**). The model’s ability to stratify patients into distinct prognostic groups remained effective, with higher risk scores still correlating with poorer OS. Moreover, we extended the validation to the entire dataset, including both the training and validation sets combined. The results from the entire dataset were similarly consistent with those observed in the training and validation sets (**Supplementary Figure S1F-J**), demonstrating that the model performs well across different subsets of LUAD patients. Patients with higher risk scores exhibited poorer prognoses across various subgroups, including age, gender, and T grade (**Supplementary Figure S2A-F**). Notably, significant differences in risk scores were observed between these subgroups, with males and patients in stages T3–4 showing markedly higher risk scores (**Supplementary Figure S2G-I**).

### Assessing immune infiltration

**Figure 4:**
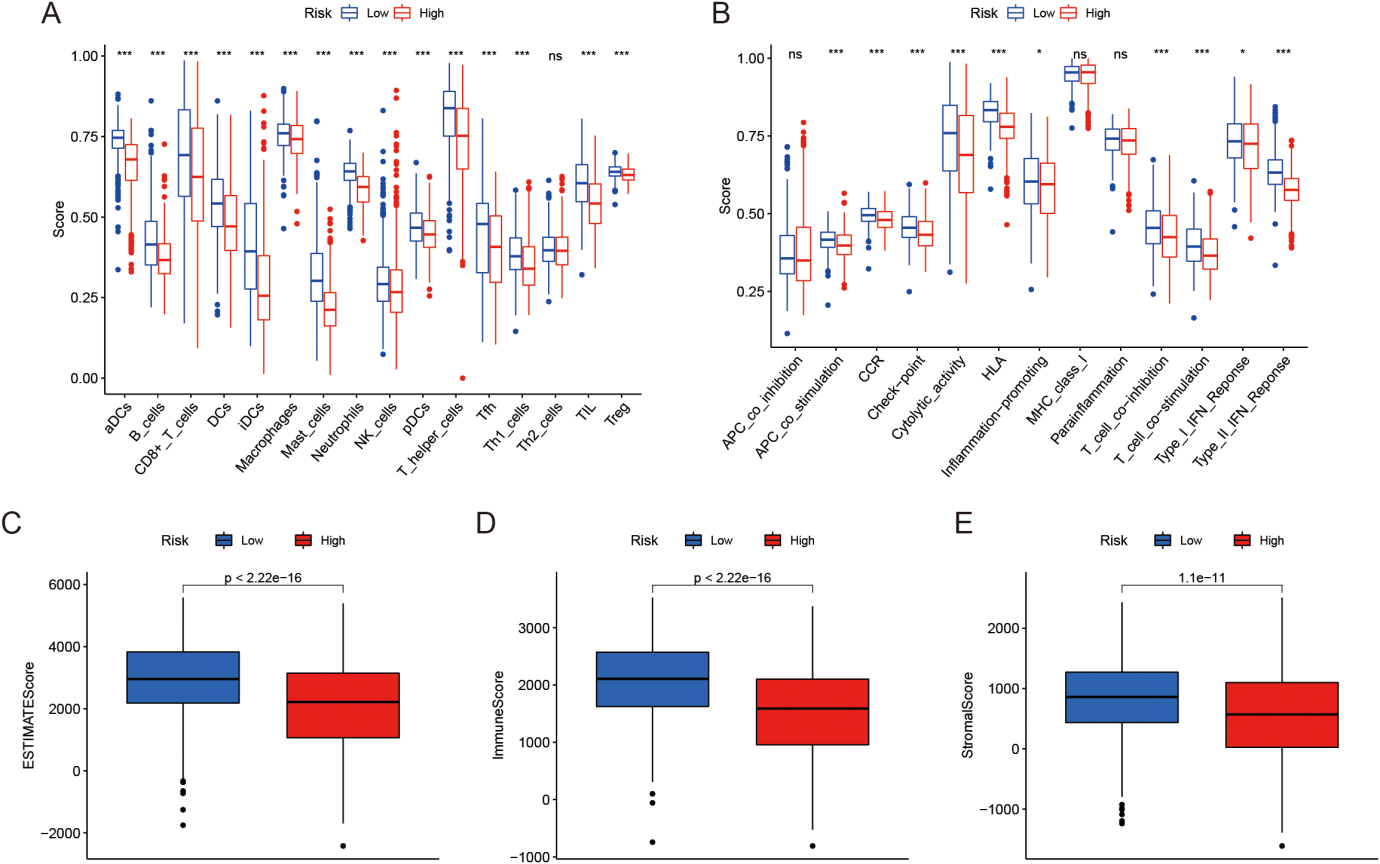
Analysis of TME and immune scores. **(A)** Distribution of infiltration of 16 immune cell types between two risk groups. **(B)** Distribution of 13 immune-related pathways between two risk groups. **(C-E)** Box plots showing the differences in Estimate score, Immune score, and Stromal score between the risk groups. *P < 0.05, ***P < 0.001, ^ns^P ≥ 0.05 compared to the control group.

We further investigated the relationship between LRGs score and TME features. Our analysis showed that the low LRGs score group had significantly higher immune cell infiltration compared to the high score group, except for Th2 cells, where no substantial difference was observed (**Figure 4A**). The increased immune cell infiltration in the low LRGs score group suggests a more active immune environment, potentially indicating a stronger anti-tumor immune response. Moreover, immune-related pathways were notably enriched in the low LRGs score group, although no significant differences were found in pathways such as APC co-inhibition, MHC class I, and Parainflammation (**Figure 4B**), pointing to a more complex involvement of these immune mechanisms in LUAD progression. Additionally, patients in the low LRGs score group showed significantly elevated ESTIMATE scores, immune scores, and stromal scores (**Figures 4C–E**). Together, these results suggest a strong association between the LRGs score and the TME in LUAD.

### Independent prognostic value of the LRGs score and development of a nomogram

**Figure 5:**
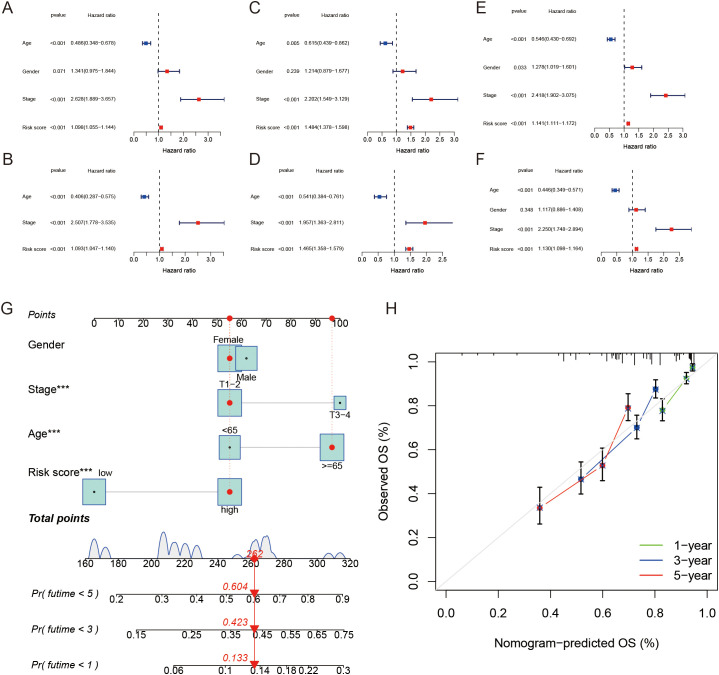
The clinical value of a nomogram for LUAD patients. **(A, B)** Forest plots presenting univariate and multivariable Cox regression analyses of clinical characteristics and risk score in the training cohort. **(C, D)** Forest plots presenting univariate and multivariable Cox regression analyses of clinical characteristics and risk score in the test cohort. **(E, F)** Forest plots presenting univariate and multivariable Cox regression analyses of clinical characteristics and risk score in the entire cohort. **(G)** Nomogram for predicting overall survival of patients based on risk score and clinicopathological factors. **(H)** Calibration plot of the nomogram showing the survival probability over time.

To assess the independent prognostic value of the LRGs score in LUAD, we performed univariate and multivariate Cox regression analyses. The univariate analysis revealed that the LRGs score, along with other clinicopathological factors such as age and tumor stage involvement, were significantly associated with OS in the training set (**Figure 5A**). Further, multivariate Cox regression analysis confirmed that the LRGs score remained an independent prognostic factor, even after adjusting for other clinical variables in the training set (**Figure 5B**). Importantly, these results were consistent across the validation set and the entire dataset (**Figures 5C–F**). To enhance the clinical applicability of the LRGs score, we constructed a prognostic nomogram by integrating the LRGs score with other significant prognostic factors identified in the multivariate analysis. It is important to note that we also assessed the association between these clinical characteristics and patient prognosis to ensure that the LRGs in the model are independently associated with the outcome. Integrating these factors together allows for a more accurate prediction of patient prognosis (**Figure 5G**). The nomogram provides a quantitative tool for estimating the 1-, 3-, and 5-year OS of LUAD patients. The calibration curves for the nomogram demonstrated good agreement between the predicted and actual survival outcomes (**Figure 5H**). These findings underscore the value of the LRGs score as an independent prognostic factor and highlight the potential of the nomogram as a practical clinical tool for personalized patient management in LUAD.

### Mutation analysis of risk model and its predictive effect on chemotherapy drug sensitivity

**Figure 6:**
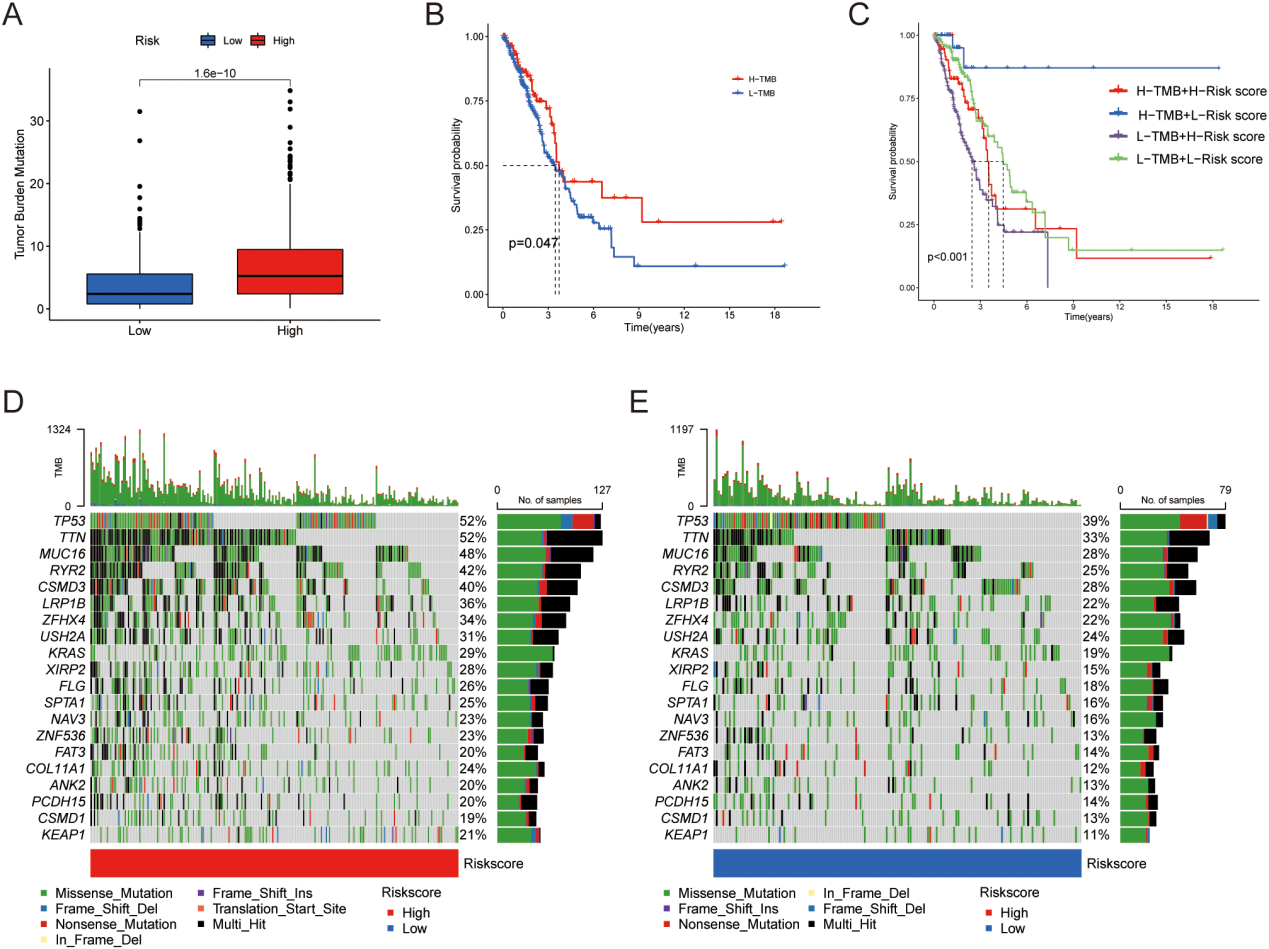
Differences in tumor mutational burden. **(A)** Distribution of TMB between the two groups. **(B)** Kaplan-Meier curves illustrating OS for patients in the high and low TMB groups. **(C)** Kaplan-Meier curves comparing OS between patients in the combined risk group and TMB groups. **(D, E)** Waterfall plot showing the top 20 mutated genes and their distribution variance between the two risk groups.

The mutation analysis of the risk model revealed distinct mutational landscapes between high- and low-risk LUAD patients. The high-risk group exhibited a higher mutation burden, as shown in **Figure 6A**. However, we found an interesting decrease in OS in patients with low tumor mutation burden (TMB) (**Figure 6B**). Survival analysis combining risk scores and TMB scores indicated that the high TMB and low-risk group had the best survival outcomes (**Figure 6C**). Next, we compared the genomic mutations between the two groups. The top five mutations in both the high- and low-risk score subgroups were similar; however, the mutation rates were generally higher in the high-risk group (**Figures 6D, E**).

**Figure 7:**
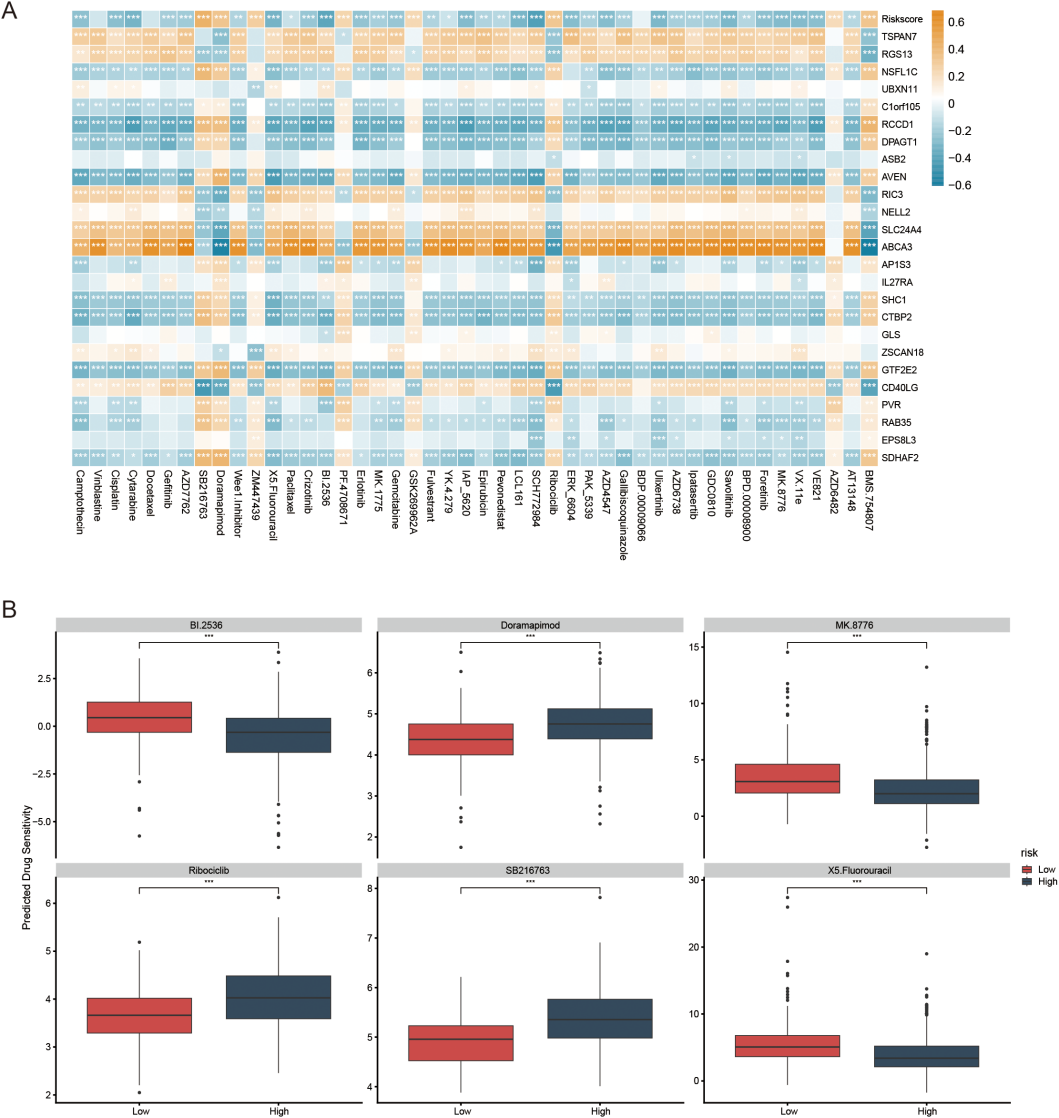
The role of prognostic models in predicting gene-drug associations and drug sensitivity. **(A)** The association between risk score, model genes, and drug sensitivity. **(B)** The boxplots illustrate the comparison of IC_50_ values between the two risk groups; *p < 0.05, **p < 0.01, ***p < 0.001.

IC_50_ analysis results showed a significant correlation between risk scores and drug sensitivity (**Figure 7A**). Specifically, patients in the low-risk group exhibited higher IC_50_ values for BI.2536, MK8776, and 5-Fluorouracil. By contrast, the high-risk group showed higher IC_50_ values for Doramapimod, Ribociclib, and SB216763 (**Figure 7B**). These findings suggest that this signature may provide valuable insights into personalized treatment regimens for LUAD patients based on their molecular subtype.

### RCCD1 promotes proliferation and migration of LUAD cells

**Figure 8:**
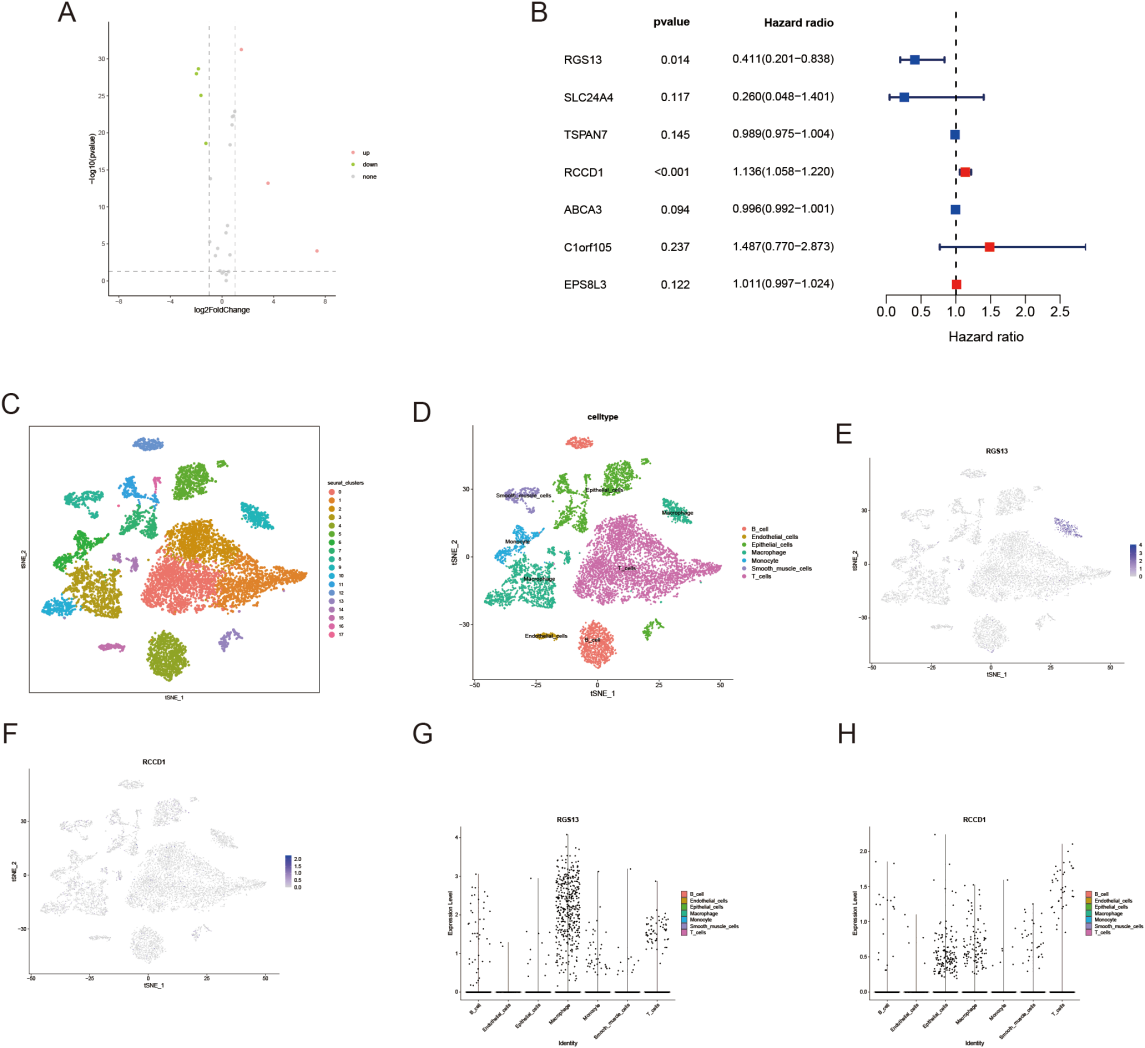
Verification of keys genes through sc-RNA seq. **(A)** The volcano plot illustrates the differential expression analysis of the core genes between tumor and adjacent normal tissue. **(B)** The forest plot demonstrates the genes that are prognostically significant for LUAD patients. **(C, D)** tSNE plots of cells generated from LUAD tissue. The plots are colored by cell cluster, and the cells are clustered into 7 sub-clusters. **(E, F)** The expression of signature genes in LUAD visualized in tSNE. **(G, H)** Violin plots depicting the expression of signature genes in clusters of LUAD. The y axis shows the normalized read count.

To further investigate the role of lactylation-related genes in LUAD progression, we performed differential expression analysis of 25 core genes in both tumor and adjacent normal tissues, which was visualized through a volcano plot (**Figure 8A**). Following univariate prognostic analysis of the selected genes, RCCD1 and RGS13 were identified as potential prognostic genes (**Figure 8B**). We then obtained single-cell sequencing data for 11 samples from GSE131907. We identified 18 distinct cell clusters (**Figure 8C**). Cell subsets were annotated using specific tools (**Figure 8D**). To investigate the expression of RCCD1 and RGS13 in different cell types, we visualized their expression using t-SNE and violin plots. RGS13 is predominantly expressed in macrophages, while RCCD1 is expressed at low levels in various cell types within the tumor microenvironment (**Figures 8E–H**).

**Figure 9:**
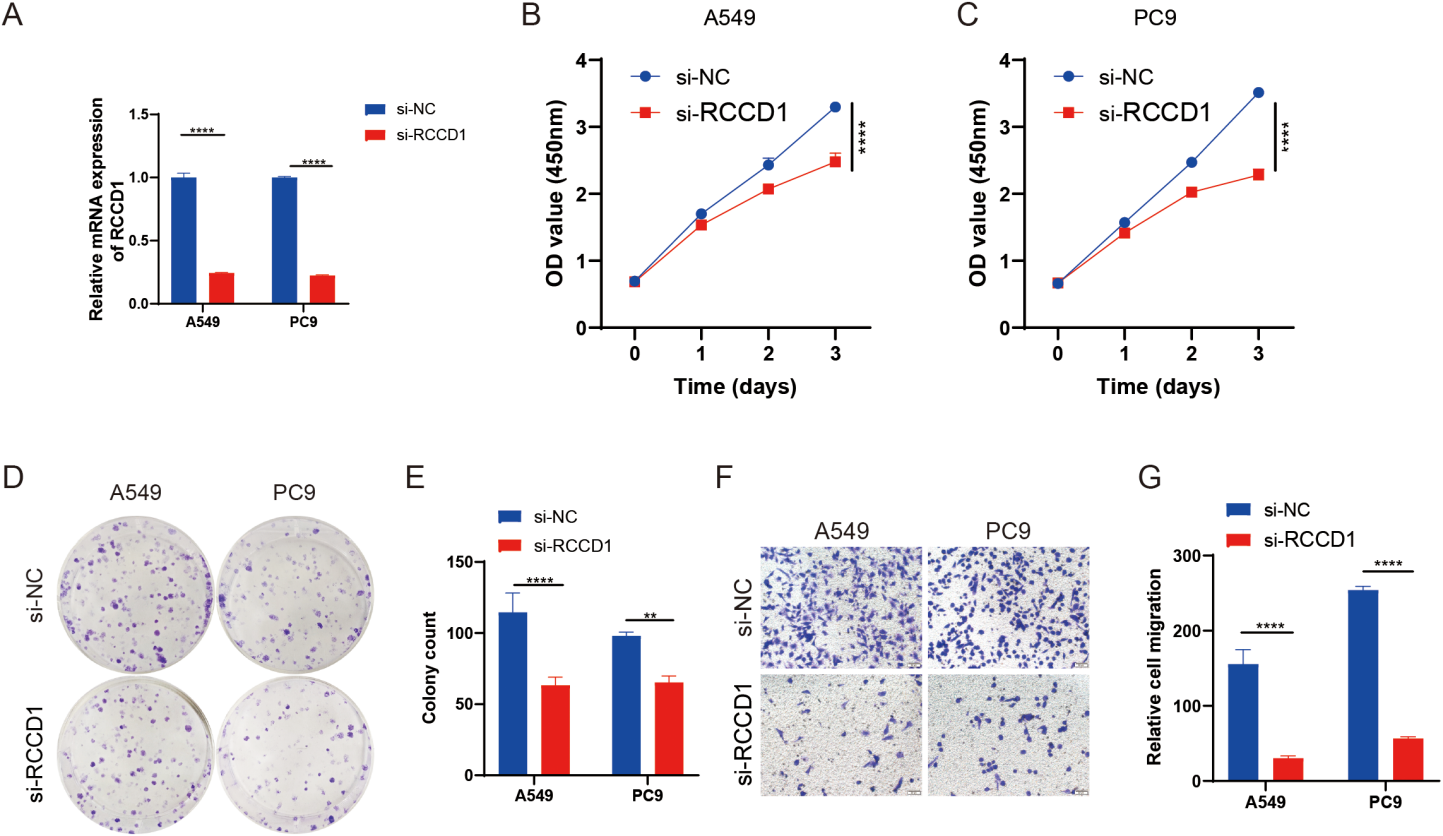
Functional analysis of RCCD1 in LUAD cells. **(A)** RCCD1 knockdown efficiency in A549 and PC9 cells measured by qRT-PCR. **(B, C)** Impact of RCCD1 knockdown on LUAD cell viability assessed by CCK-8 assays. **(D, E)** Effect of RCCD1 knockdown on colony formation in LUAD cells assessed by colony-forming assay. **(F, G)** Effect of RCCD1 knockdown on cell migration in LUAD cells measured by transwell assay. **P < 0.01, ****P < 0.0001 compared to the control group.

The effectiveness of knockdown was confirmed by RT-qPCR (**Figure 9A**). Further experiments showed that knockdown of RCCD1 in LUAD cell lines (A549 and PC9) led to a significant reduction in cell proliferation and migration, as confirmed by CCK-8 assays (**Figures 9B, C**), colony formation assays (**Figures 9D, E**), and Transwell migration assays (**Figures 9F, G**). These findings suggest that RCCD1 promotes LUAD cell proliferation and migration, potentially through the modulation of lactylation-related pathways. Our results indicate that RCCD1 plays a crucial role in LUAD progression by enhancing cell proliferation and migration.

## Discussion

This study provides an integrative analysis of lactylation-related gene signatures in LUAD and their association with immune cell infiltration. By combining scRNA-seq and bulk RNA sequencing datasets, we identified key lactylation-related genes with significant dysregulation in LUAD and demonstrated their potential roles in tumor progression and immune modulation. Notably, we found that lactylation-related genes are closely associated with immune cell infiltration, suggesting that these genes may play important roles in the TME and immune responses. Additionally, survival analysis revealed that lactylation-related gene signatures serve as independent prognostic biomarkers for LUAD, with high expression levels correlating with poor OS, indicating their potential application in predicting clinical outcomes in LUAD patients. However, further experimental studies are needed to elucidate the precise molecular mechanisms underlying these observations, especially the immune regulatory roles and regulatory networks of lactylation-related genes in LUAD.

In our analysis, RCCD1 emerged as a core gene identified as a potential oncogene and closely associated with LUAD progression. RCCD1 participates in cellular stress responses and metabolic regulation, is highly expressed in LUAD tissues, and correlates with poor prognosis (24, 25). Functional studies revealed that knocking down RCCD1 in LUAD cell lines (A549 and PC9) significantly reduced cell proliferation and migration. These findings suggest that RCCD1 may promote LUAD cell proliferation by modulating lactylation-related pathways and metabolic reprogramming. Regarding immune regulation, the role of RCCD1 in immune cell infiltration and polarization warrants further investigation. Given involvement of RCCD1 in metabolic processes and its potential in regulating immune responses, it may serve as a promising therapeutic target for future interventions aimed at reprogramming the TME and enhancing antitumor immunity. Although this study relies on publicly available data and primarily focuses on correlation analysis, it provides critical insights into the potential of lactylation-related genes as novel diagnostic and therapeutic targets. Targeting these pathways could disrupt the metabolic and immunosuppressive mechanisms in the TME, and combining lactylation-targeted therapies with immune checkpoint inhibitors or metabolic modulators may enhance antitumor immunity and improve clinical outcomes for LUAD patients.

In conclusion, this study emphasizes the importance of lactylation-related genes, especially RCCD1, in LUAD progression. These findings lay the foundation for future research into the clinical and therapeutic applications of lactylation-related genes, advancing the development of personalized cancer treatment strategies.

## Data Availability

The datasets presented in this study can be found in online repositories. The names of the repository/repositories and accession number(s) can be found in the article/**Supplementary Material**.
