# Supplementary material for: A novel lactylation-related gene signature to predict prognosis and treatment response in lung adenocarcinoma
Source: Front Oncol. 2025 Mar 14;15:1549724. doi: 10.3389/fonc.2025.1549724 (PMC11949803; doi:10.3389/fonc.2025.1549724)
Supplement: Supplementary file 1 [file DataSheet1.docx]

**Supplementary Materials**

Supplementary figures


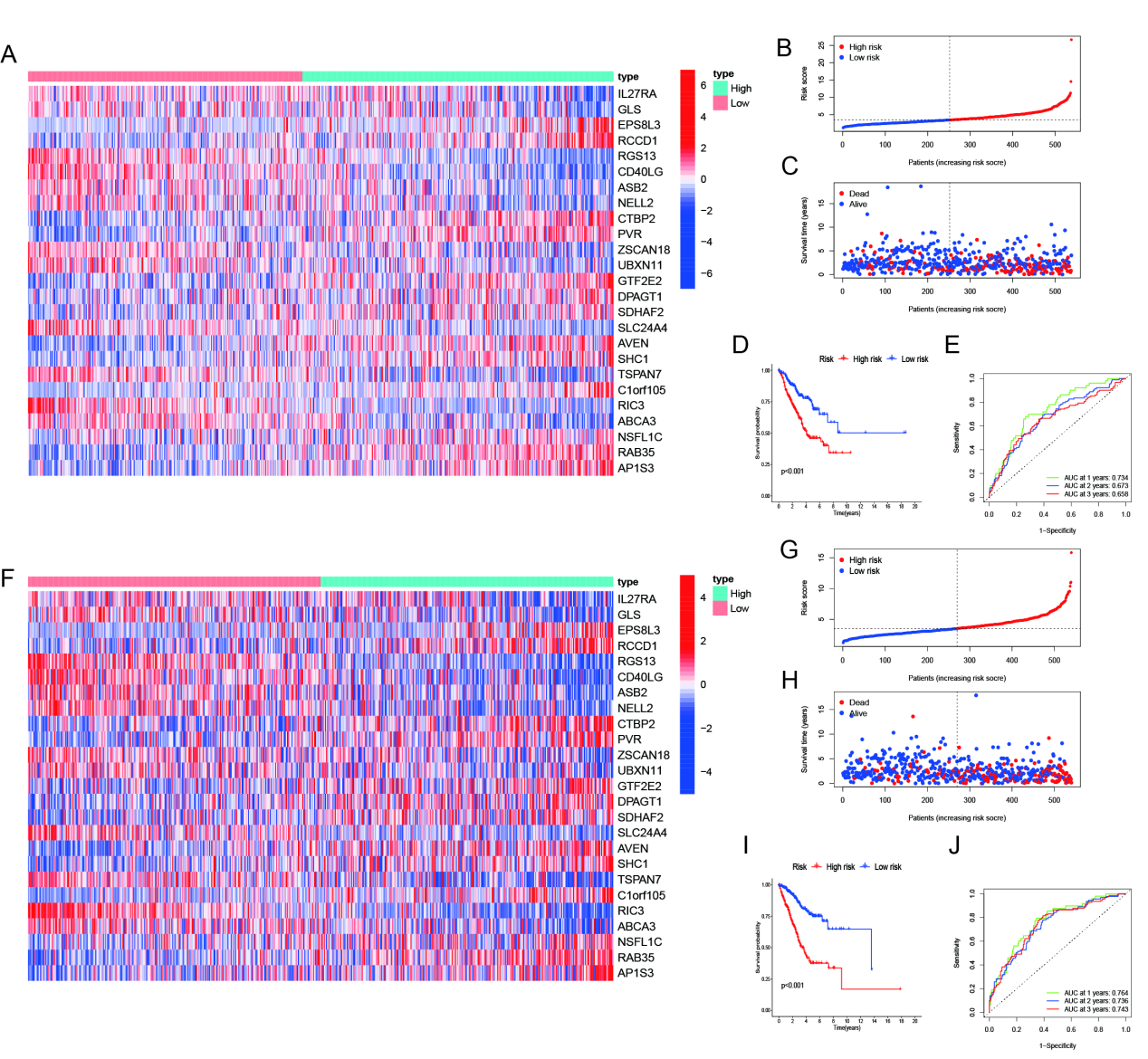


Figure S1: Validation of the 25-genes signature in the test and entire cohort. (A) Heatmap of the test set illustrating the expression of selected genes in different risk subgroups. (B, C) Distribution of risk scores based on survival status and time for the test set. (D) Kaplan-Meier survival curves showing overall survival for patients categorized into different risk groups. (E) ROC curves evaluating the sensitivity and specificity for 1-, 2-, and 3-year survival predictions in the test set. (F) Heatmap of the entire set displaying the expression of selected genes in various risk subgroups. (G, H) Distribution of risk scores based on survival status and time for the entire set. (I) Kaplan-Meier curves depicting OS for patients in the different risk groups. (J) ROC curves assessing sensitivity and specificity for 1-, 2-, and 3-year survival predictions in the entire set.


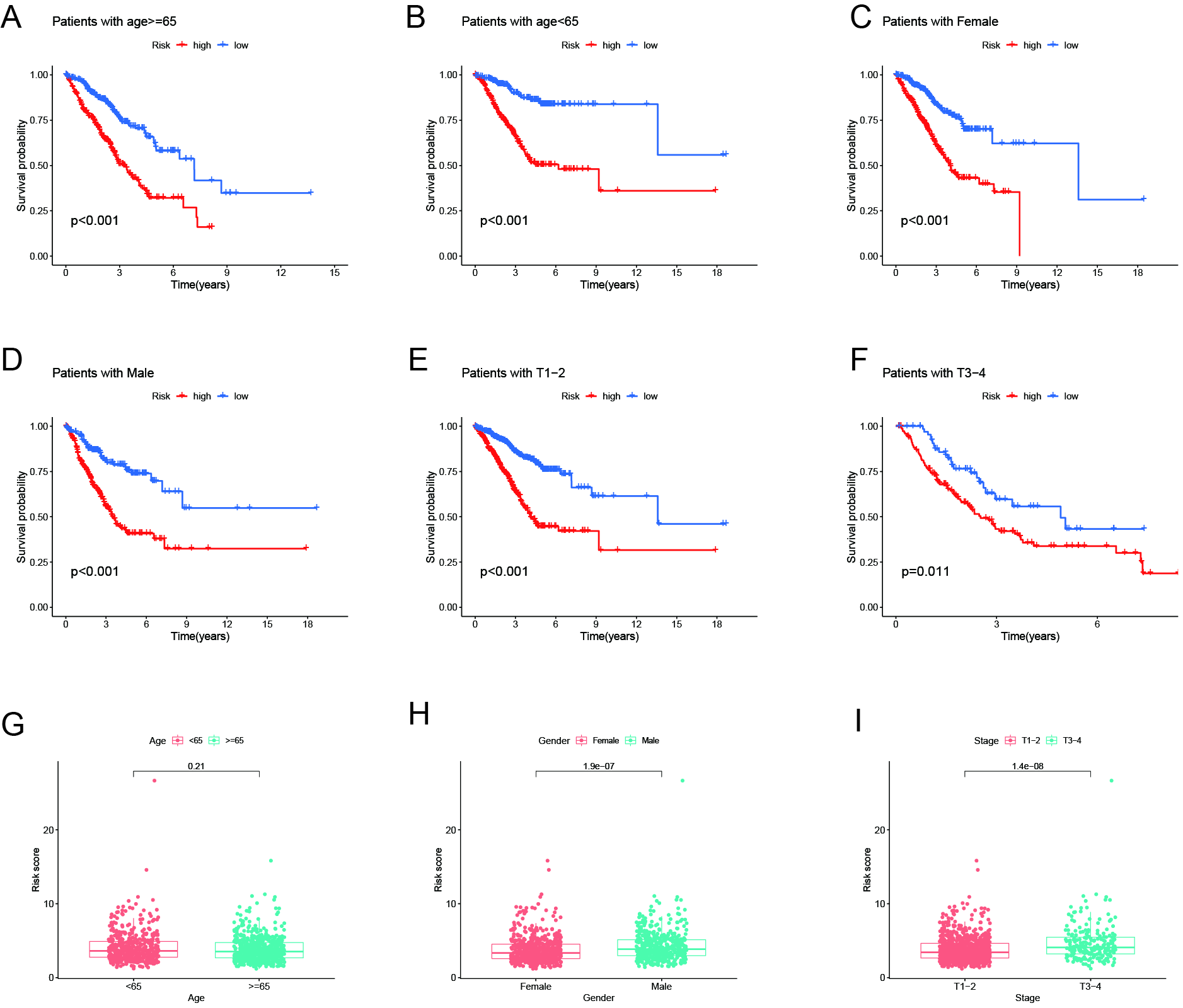


Figure S2: Differences in survival and risk scores across subgroups. (A, B) Kaplan-Meier survival curves illustrating survival outcomes of patients aged ＜65 and ≥ 65 years within different risk score groups. (C, D) Kaplan-Meier survival curves illustrating survival outcomes of female and male patients within different risk score groups. (E, F) Kaplan-Meier survival curves illustrating survival outcomes of patients with pathological stages T1-2 and T3-4 within different risk score groups. (G-I) Box plots showing the distribution and differences in risk scores across different age groups, gender groups, and T stage groups.

Supplementary Tables

Table S1. The clinical characteristics of the TCGA，GSE31210 and GSE72094 cohorts.

| Clinical characteristic | | TCGA set | GSE31210 set | GSE72094 set |
| --- | --- | --- | --- | --- |
| Age | <65 | 215 | 164 | 107 |
|  | ≥65 | 254 | 62 | 291 |
| Gender | Male | 213 | 105 | 176 |
|  | Female | 256 | 121 | 222 |
| Stage | Ⅰ-Ⅱ | 367 | 226 | 321 |
|  | Ⅲ-Ⅳ | 102 | 0 | 72 |
|  | Unknown |  |  | 5 |

Table S2. Summary of Lactylation-related genes.

| **Lactylation-related genes** | | | | |
| --- | --- | --- | --- | --- |
| ACAT2 | EEF1G | HMGN2 | PAK2 | SATB1 |
| ACIN1 | EEF2 | HMGN3 | PARP1 | SET |
| ADAR | EHMT2 | HMGN4 | PCBP1 | SF3A1 |
| ADNP | EIF3D | HNRNPA1 | PCBP2 | SF3B1 |
| AHNAK | EIF3J | HNRNPC | PCMT1 | SFPQ |
| ALB | EIF4G1 | HNRNPD | PCNP | SFPQ |
| ALDH1A1 | EIF4G2 | HNRNPF | PDAP1 | SH3GL1 |
| ALDOA | EIF4H | HNRNPH1 | PDLIM1 | SIRT1 |
| ALDOB | EMG1 | HNRNPK | PES1 | SIRT2 |
| ALYREF | ENO1 | HNRNPL | PFKM | SIRT3 |
| ARGLU1 | ENSA | HNRNPM | PFKP | SMAP |
| ARID1A | EP300 | HNRNPU | PFN1 | SMARCA5 |
| ARID3A | FABP5 | HSDL2 | PGK1 | SMARCC1 |
| ARID3B | FAM50A | HSPE1 | PHC3 | SMARCC2 |
| ARPP19 | FKBP3 | IARS2 | PHF6 | SMC3 |
| BCLAF1 | FLYWCH2 | IFI16 | PKM2 | SNRPA1 |
| BOLA2 | FUBP1 | IK | POLDIP3 | SOD1 |
| BRD4 | G6PD | IKZF1 | PPIA | SPR |
| BTF3 | GAPDH | ILF2 | PPIL4 | SPR14 |
| BZW1 | GATAD2A | ILF3 | PPM1G | SRP14 |
| BZW2 | GATAD2B | IRF2BP2 | PPP1CB | SRRM1 |
| C19orf53 | GFAP | JMJD1C | PPP1CC | SRRM2 |
| CACYBP | GIGYF2 | JPT1 | PPP1R2B | SSB |
| CALD1 | GTF2F1 | JPT2 | PRAM1 | STMN1 |
| CALM1 | GTF2I | KHDRBS1 | PRCC | SUB1 |
| CALML5 | H1-2 | KHSRP | PRDX1 | SUMO2 |
| CALR | H1-3 | KIF2C | PRKDC | TCOF1 |
| CBR1 | H1-5 | KRT1 | PRPF6 | TERF2 |
| CBX3 | H2AFV | KRT10 | PSMA7 | THOC2 |
| CBX5 | H2AFZ | LAP3 | PSMC1 | THRAP3 |
| CCNA2 | H2AJ | LCP1 | PSME3IP1 | THUMPD1 |
| CCT5 | H2AX | LDHB | PTBP1 | TKT |
| CD2BP2 | H2AZ1 | LEMD3 | PTMA | TMA7 |
| CDC5L | H2AZ2 | LGALS1 | RACGAP1 | TMPO |
| CDV3 | H2BC13 | LRPPRC | RALYL | TMSB4X |
| CDV3 | H2BC14 | LSP1 | RAN | TOP2B |
| CDYL | H2BC18 | MAGOH | RANBP2 | TP53 |
| CEBPZ | H2BC5 | MAGOHB | RB1 | TPM4 |
| CFDP1 | H2BU1 | MAP2K4 | RBM10 | TPR |
| CHD4 | H3-3A | MAPRE1 | RBM14 | TRIM28 |
| CHERP | H3C1 | MBD2 | RBM17 | TRIR |
| CNN2 | H3C15 | MBP | RBM25 | TSSC4 |
| CNN3 | H4C1 | MDC1 | RBM39 | U2AF2 |
| COPS4 | HCFC1 | MKI67 | RBMX | U2SURP |
| CRABP2 | HDAC1 | MNDA | RCC2 | UBE2E1 |
| CSRP1 | HDAC2 | MPHOSPH6 | RECQL | UBE2M |
| CSRP2 | HDAC3 | MSN | RFC1 | UPF1 |
| CWC15 | HDGF | MTA1 | RFC4 | VARS |
| DDX17 | HDGFL2 | MTA2 | RIMS1 | VIM |
| DDX18 | HEXIM1 | MYH13 | RPA1 | WAS |
| DDX21 | HIST1H1C | NCDN | RPL13 | WBP11 |
| DDX39A | HIST1H2BB | NCL | RPL14 | WDR33 |
| DDX39B | HIST1H2BD | NEFL | RPL22 | WIZ |
| DDX3X | HIST1H2BH | NHLRC2 | RPL24 | XPO5 |
| DDX41 | HIST1H2BK | NOC3L | RPL29 | XRCC4 |
| DDX42 | HIST1H2BL | NOLC1 | RPL5 | YLPM1 |
| DDX46 | HIST1H2BN | NONO | RPS11 | ZC3H18 |
| DDX5 | HIST1H2BO | NOP2 | RPS23 | ZC3H4 |
| DECR1 | HIST1H3A | NPM1 | RPS27A | ZMYM3 |
| DFFA | HIST1H4A | NSUN2 | RRP1B | ZNF207 |
| DHRS7 | HIST2H2BE | NUCKS1 | RSL1D1 | ZNF280C |
| DHX16 | HIST2H2BF | NUDT21 | S100A11 | ZNF706 |
| DHX9 | HISTIH2BH | NUDT5 | S100A4 | ZNFX1 |
| EAF1 | HLTF | NUP133 | S100A6 | ZRANB2 |
| ECHDC1 | HMGA1 | NUP50 | SAFB | ZYX |
| EDF1 | HMGB1 | PABPC1 | SARNP | PABPN1 |
| EEF1A1 | HMGN1 |  |  |  |
